# A Photonic Switch Based on a Hybrid Combination of Metallic Nanoholes and Phase-change Vanadium Dioxide

**DOI:** 10.1038/s41598-018-29476-6

**Published:** 2018-07-23

**Authors:** Miao Sun, Mohammad Taha, Sumeet Walia, Madhu Bhaskaran, Sharath Sriram, William Shieh, Ranjith Rajasekharan Unnithan

**Affiliations:** 1 Electrical & Electronic Engineering Department, University of Melbourne, Parkville, 3010 Australia; 2 Functional Materials and Microsystems Research Group and the Micro Nano Research Facility, RMIT University, GPO Box 2476, Melbourne, Victoria 3001 Australia

## Abstract

A photonic switch is an integral part of optical telecommunication systems. A plasmonic bandpass filter integrated with materials exhibiting phase transition can be used as a thermally reconfigurable optical switch. This paper presents the design and demonstration of a broadband photonic switch based on an aluminium nanohole array on quartz utilising the semiconductor-to-metal phase transition of vanadium dioxide. The fabricated switch shows an operating range over 650 nm around the optical communication C, L, and U band with maximum 20%, 23% and 26% transmission difference in switching in the C band, L band, and U band, respectively. The extinction ratio is around 5 dB in the entire operation range. This architecture is a precursor for developing micron-size photonic switches and ultra-compact modulators for thin film photonics.

## Introduction

Photonic switches are devices used in optical communication and computing network that can establish or release the connection of optical signals^1^. There is a huge demand for ultra-compact photonic switches because of the rapid advancements in the high-data-rate fibre-optic communication systems, and high-speed optical computing systems^2,3^.

The switching operation in an optical domain can be achieved by opto-mechanism^4^, acousto-optic^5^, magneto-optic^6^, or electro-optic methods^7–9^. Photonic switches, where thermal energy is used for changing electro-optics properties of the switch, are called thermally reconfigurable photonic/optical switches. Thermally reconfigurable optical switches have several advantages such as easy fabrication, structural simplicity and ample choices of a thermo-optic functional materials^10^. However, thermally reconfigurable photonic switches are bulky and hence integration with state-of-the-art electronics is a challenge.

Plasmonics offers an attractive platform to bridge the size mismatch between optical devices and electronics, and hence enable compact integration of these devices on a single chip^11^. Surface plasmons (SP) are free electron oscillations propagating at a metal-dielectric interface, accompanied by electromagnetic oscillations^12^. Enhanced localization of electric field can be achieved by using the plasmonics, and this effect allows for the development of nanoscale-optic devices beyond the diffraction limit^13–16^. Nanoscale devices based on plasmonic metamaterials attract keen interest for the development of next-generation optoelectronic devices^17,18^, including metasurface filters^19–22^, metamaterials switches and modulators^23^.

Plasmonic wavelength filters based on perforated metallic film integrated with suitable materials exhibiting phase transition can be used as a thermally reconfigurable photonic switch operate in submicron scale^24,25^. One such material is vanadium dioxide (VO_2_), a canonical Mott material with a large refractive index change from 3.24 + 0.30*i* to 2.03 + 2.64*i* at 1550 nm during semiconductor-to-metal (semi-metal) phase transition^26^. VO_2_ has been extensively explored in recent years because of its large refractive index change during the phase transition^27^. The phase transition can be triggered by thermal heating in milliseconds, light irradiation in femtoseconds or external electrical field in picoseconds^28–31^. Combining submicron light confinement of plasmonics with large refractive index change of VO_2_ can be exploited for making novel photonic devices. Recently, versatile applications of plasmonics by utilizing VO_2_ phase transition have been explored, such as metasurfaces^24,32^, optical memory device^31^ nanoscale antenna^33^, temperature sensor^34^, rewriteable devices^35^, and ring modulator^36^. For electro-optical applications, the precise control of VO_2_ phase transition is essential^37,38^. A switchable metasurface based on VO_2_ working at THz region is recently proposed based on computer simulations which can be switched from broadband absorber to a reflecting brandband halfwave plate by temperature tuning^39^. VO_2_ – Ag thermal waveguide switch is experimentally demonstrated with a typical 50% roll-off frequency of 25 kHz in 10 um waveguide length^39^. The thermal waveguide switch gives an idea of switching time which is around microsecond using thermal trigger for the plasmonic VO_2_ switch^40^. Recently, VO_2_ - Ag and VO_2_ – Au based thermally-driven optical switches operating near IR region (800 nm to 900 nm) have been demonstrated with 4% and 1.2% transmission respectively using a square arrangement of holes^41^.

In this manuscript, we present, using both computational and experimental methods, a broadband photonic switch based on a hybrid combination of a hexagonal array of holes and vanadium oxide. The hexagonal arrangement of holes in aluminium is fabricated on a quartz substrate, followed by the deposition of VO_2_ on the nanohole array. The proposed geometry has several advantages. The fabricated photonic switch is polarization independent due to the hexagonal arrangement of holes. A hexagonal arrangement will also increase the holes per area and increase the efficiency of the device. Aluminium is CMOS compatible and inexpensive compared to gold and silver. A high transmission of 37.5% at optical communication region is achieved in the hole array with an optimized device geometry and a thin layer of VO_2_ with a thickness of 25 nm atop hole array. The switching of the plasmonic hole array is achieved thermally by changing the refractive index of VO_2_ that results in a 21% transmission change with 4.3 dB extinction ratio in the C band, 23% transmission change with 5 dB extinction ratio in the L band, and 26.5% transmission change with 5.3 dB extinction ratio in the U band with an operating range over 650 nm. It is for the first time that such a combination of the hexagonal array with semiconductor-to-metal phase transition of VO_2_ has been explored for the fabrication of a thermally reconfigurable photonic switch, resulting in such high transmission efficiency in the optical communication range.

## Device Design and Simulation

**Figure 1:**
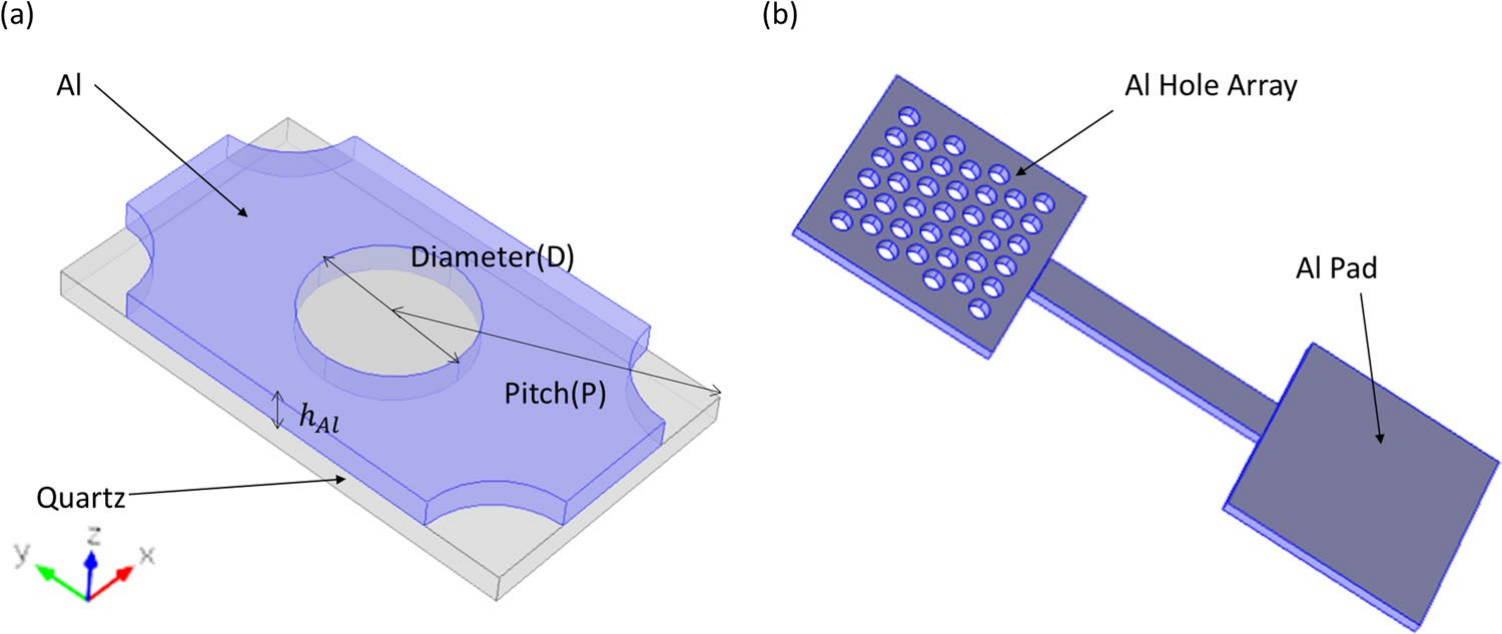
(**a**) Simulation model of a hexagonal arrangement of holes in aluminium. The peak wavelength is tuned to 1550 nm with pitch (P) = 1010 nm, hole diameter (D) = 560 nm and aluminium thickness of 100 nm. (**b**) Schematics of the device with hexagonal nanohole array in aluminium along with extended metal pad for heating. (The top VO_2_ layer is not shown).

Ebbesen reported in 1998^42^ the extraordinary optical transmission in a thin metal film when the hole diameter is under the cutoff of the first propagating mode. The dominant resonant surface plasmon excitation leads to a wavelength selection with 1000 times higher transmission than the prediction of conventional aperture theory^43^. This extraordinary optical transmission has been observed in noble metal thin films like Ag, Au, Cu, as well in transition metals like Co, Ni, W^44^, over a wide range of frequencies^45–47^. Ever since, extensive research has been conducted on structures with periodic roughness like nanoparticles, grooves, and arrays using subwavelength holes^48^. The subwavelength hole arrays in thin metallic film exhibit the character of optical filters with enhanced transmission, which has been widely applied, and the major contributions are focused on biomolecular sensors^49^, nano-antennas^50^, plasmonic optical filters^51^, plasmonic modulators and switches^41,52^. For the hole array based filters that are operating in transmission mode, resonant peaks in transmission are dominated by the surface plasmon mode excited at the cylindrical hole boundaries and two facets. In a triangular (hexagonal) hole array based plasmonic filter, the transmission peaks can be approximately predicted using the equation (1) given by^53^

$${\lambda }_{max}=\frac{a}{\sqrt{\frac{4}{3}({i}^{2}+ij+{j}^{2})}}\sqrt{\frac{{\varepsilon }_{m}{\varepsilon }_{d}}{{\varepsilon }_{m}+{\varepsilon }_{d}}},$$

where $a$ is the pitch of the array, *ε*_*m*_ and, *ε*_*d*_ refer to the dielectric constants of metal and dielectric material, and $\,i,j$ refer to scattering order. The *λ*_*max*_ actually is the minimum transmission of wavelength right before the resonance peak at longer wavelength. The scattering orders can be considered to be *i* = 1, *j* = 0, if only the first transmission minimum before the peak is considered. Using the above equation, it is possible to optimize the hole array filter to any wavelength of interest. Detailed optimization of the filter to operate at optical telecommunication range was carried out using finite element method implemented in COMSOL Multiphysics. The simulation mesh size has been adjusted according to the maximum computing power available in our lab. The simulation model consists of 100 nm thick layer of aluminium on a semi-infinite glass substrate with the refractive index value, 1.45. The refractive indices of aluminium for different wavelengths were taken from Johnston and Christy^54^. Top view of a section of simulation model is shown in Fig. 1(a), which shows quartz substrate and 100 nm thick aluminium along with hole geometry (without top layer of VO_2_). The resonance peak is finely tuned to 1.55 um by varying the pitch of hole array to 1010 nm (P = 1010 nm) and keeping hole diameter (D = 560 nm) and the thickness of aluminium (100 nm) constant. A 10-nm Chromium layer is inserted beneath the Al film as an adhesion layer using transition boundary condition. The refractive indices are taken from Johnston and Christy^55^. The transition boundary condition is used for simulating ultra-thin layers with thickness hard to be covered by mesh size of the simulation model. A VO_2_ layer was added on the top of aluminum layer with holes filling the VO_2_ to make the photonic switch. The thickness of VO_2_ was varied from between 100 nm and 25 nm (using transition layer/transition boundary condition) to study its effect on transmission and switching. A 500-nm air superstrate is constructed on the top of VO_2_ (not shown in Fig. 1(a)). To simulate a large hexagonal array, periodic boundary condition was applied on four sides of the model as shown in Fig. 1(a). The incident light was set to propagate along the z-axis (perpendicular to the surface of hole array, x-y plane) with TE polarization using port boundary condition. A 400-nm Perfect match layer (PML) is added to the top boundary and the bottom boundary to absorb the outgoing wave and to ensure that no reflection goes into the interior region. Figure 1(b) shows the schematics of the photonic switch with hole array deposited with VO_2_ along with an extended metal pad for heating VO_2_ to change its phase. The Al pad is used for attaching heater for characterizing the transmission and switching of the photonic switch for different temperature values.

**Figure 2:**
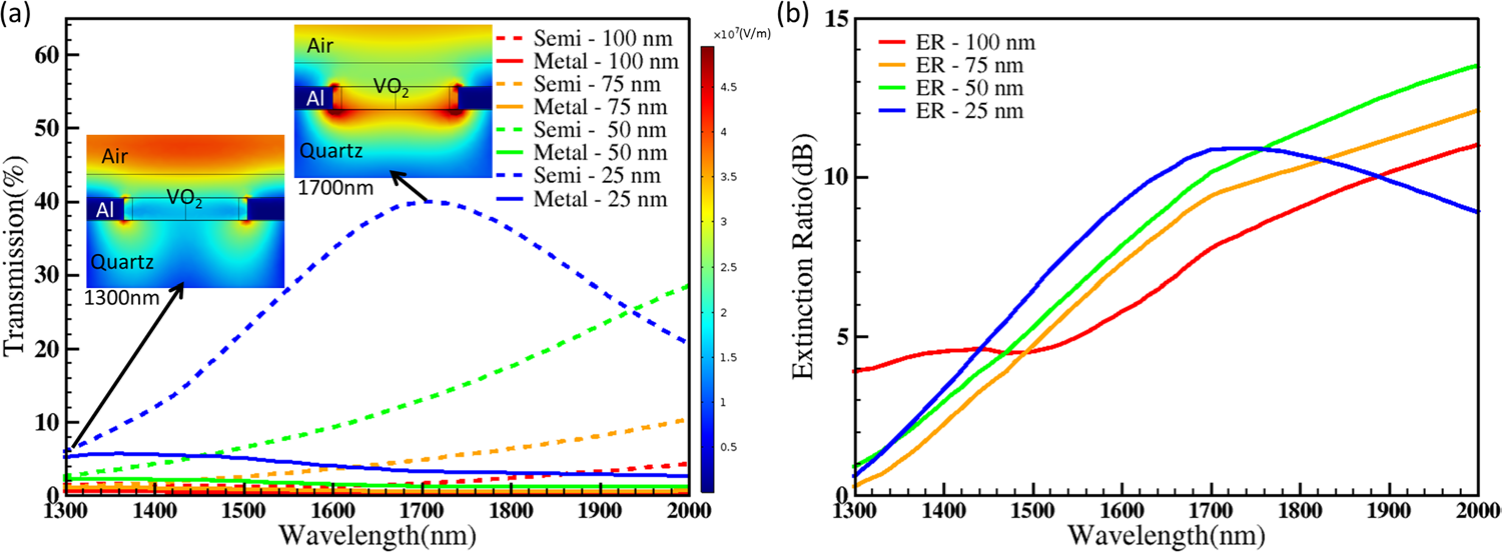
(**a**) Simulated spectrum of the photonic switch made of aluminium nanohole array and VO_2_ for semiconductor (dash line) and metallic phases (solid line) with 100-nm, 75-nm, 50-nm and 25-nm thickness VO_2_ (simulation parameters used: thickness of aluminium: 100 nm, diameter of holes: 560 nm, pitch of holes: 1010 nm, and thickness of VO_2_: 100 nm/75 nm/50 nm/25 nm). Two insets show the electrical field distribution of the cross-section of photonic switch with 25-nm VO_2_ in semiconductor phase at wavelength 1300 nm and 1700 nm respectively. The color legend of the insets is shown on the right hand side. (**b**) Extinction Ratio (ER) of the switch with respect to wavelength showing its switching ability and broad working wavelength with 100-nm, 75-nm, 50-nm and 25-nmVO_2_ respectively.

Figure 2(a,b) show the simulated transmission spectrum and extinction ratio of the above photonic switch optimized to operate in telecommunication wavelength for both semiconducting and metallic phases of VO_2_ with different thickness 100 nm, 75 nm, 50 nm and 25 nm. The 2 insets of Fig. 2(a) show electrical field distributions on the cross-section of the device with 25-nm VO_2_ in semiconductor phase at wavelengths of 1300 nm and 1700 nm respectively. The results show that the device work in the reflection mode with more E-field energy reflected and concentrated on the super-substrate air rather than passing through at 1300 nm, whereas in transmission mode the E-field energy concentrated in the cylindrical waveguide and transmitted through the substrate quartz at 1700 nm. The simulation results are in agreement with detailed mode analysis discussed in the prior art^56^. The transmission and the extinction obtained in C, L, and U band are given in Table 1. The wavelength was swept from 1300 nm to 2000 nm.

**Table 1:** ^*^The transmission difference (*TD*) is difference between the transmission of semiconductor phase and metallic phase of VO_2_.

Thickness_VO_2_[nm]	*TD*_*C*_[%]	*TD*_*L*_[%]	*TD*_*U*_[%]	*ER*_*C*_[dB]	*ER*_*L*_[dB]	*ER*_*U*_[dB]
100	0.8	1	1.3	5.1	6.2	7.3
75	2.4	3.3	4.0	6.3	8	9
50	6	8.5	11	7	8.3	9.7
25	25	32	36.5	8.2	9.6	10.6

The transmission difference and extinction ratio in C, L, and U band for different thickness of VO_2_ are taken from Fig. 2. **TD**_**C**_, **TD**_**L**_, **TD**_**U**_: corresponds to maximum transmission difference in C, L and U bands; ***ER***_***C***_, ***ER***_***L***_, ***ER***_***U***_: corresponds to the maximum extinction ratio in C, L and U band.

It was found that as the VO_2_ film thickness decreases, there is an increase in the extinction ratio and transmission. However, practically it is difficult to reduce the thickness of VO_2_ to less than 25 nm by keeping the uniformity of the film, especially filling on the Al nanoholes without creating patches. Hence there is a trade-off between the feasibility of fabrication and device performance. For the photonic switch, the thickness of VO_2_ was selected to be 25 nm to increase the transmission efficiency of C, L and U band (example, 3.4% and 39.5% transmission in the metallic and semiconducting phase of VO_2_ with extinction ratio 10.6 dB at 1675 nm as shown in Fig. 2). The results also show that the switch operates in a broad wavelength range from 1530 nm to 2000 nm with less than 3-dB loss with 25-nm VO_2_. This ensures that the photonic switch covers the wavelength window used in optical communications. These results also show that the device can act as a switch by changing the phase of VO_2_ from semiconducting (ON state) to metallic state (OFF state) with the application of heat (68 °C).

## Device Fabrication and Characterization

**Figure 3:**
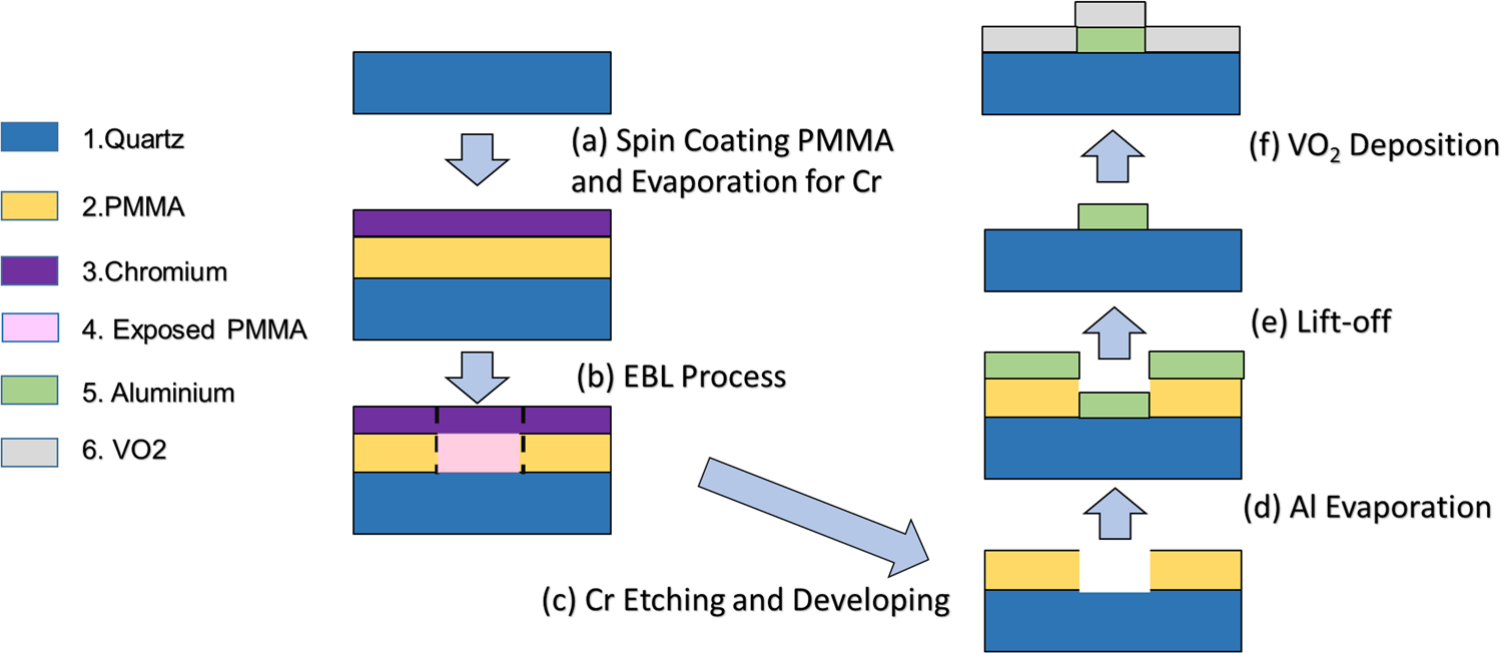
Fabrication process flowchart of the photonic switch with the material legend shows on the left. The light blue arrow shows the fabrication flow direction from step (a) to step (f). The material legend shows on the left-hand side.

Based on the above simulation results, photonic switches were fabricated on a 500 µm thick quartz substrate using electron beam lithography (EBL) (Vistec EBPG5000plusES). The fabrication steps are shown in Fig. 3 (steps (a)–(f)). In the first step (a), a 350 nm thick double layer of PMMA(Polymethyl methacrylate) was spun on the quartz substrate by Pico Track PCT-150RRE, followed by depositing a 30 nm Cr on the top of PMMA using Electron beam evaporation (EBPVD: Intlvac Nanochrome II) in order to have a conducting surface for EBL patterning. In the second step (b), EBL was used to write the nanohole array pattern on PMMA. Before developing the patterned sample, the conductive layer of Cr was removed by wet etching. This is followed by developing the sample into a mixer of MIBK (Methyl isobutyl ketone) and IPA to make holes in PMMA as shown in step (c). Following this, a 100-nm aluminium thin film was deposited using EBPVD (step (d)) followed by lift off in step (d). Prior to the Al metallization, the substrate was coated with 10 nm Chromium as adhesion layer within the same deposition step. The fabricated hole array is expected to have a hole diameter of 560 nm and pitch of 1010 nm. Extra metallic pad made of aluminium was connected to the hole array for heating the device shown in Fig. 1(b). In the final step (f), VO_2_ is deposited on top of the aluminium. A quartz substrate is cleaned and plasma treated in an argon environment to enhance adhesion between the VO_2_ film and the substrate. VO_2_ is deposited using the pulsed-DC magnetron sputtering technique. A Vanadium (99.99%) target is used in for sputtering. The sputtering chamber is allowed to reach 4.0 × 10^−7^ Torr before the introduction of the Ar: O_2_ gas mixture. Ar: O_2_ mixtures is introduced with a flow rate of 12.25:5.25 sccm respectively (for 30% O_2_). Sputtering is done at 2.8 × 10^−3^ Torr pressure, a power of 200 W with 25 kHz pulse frequency and 5 µs reverse time. Deposition is done for 45 minutes at room temperature producing amorphous VO_2_. Subsequently, the as-deposited VO_2_ films are annealed in a furnace, evacuated to low vacuum to achieve a pressure of ~250 mTorr, at 550 °C for 90 min. Post-deposition annealing at low pressure enhances the level of control over oxygen vacancies and limits oxygen loss, which happens at a rapid rate in VO_2_ thin films. X-ray photoelectron spectroscopy (XPS), X-ray diffraction (XRD), and micro-Raman spectroscopy were conducted to characterize the VO_2_ thin films^57^. The thin films showed good insulator–metal transition, as expected at ~68 °C for the VO_2_ phase of vanadium oxide^57^. This allows the formation of excellent VO_2_ thin films on top of Al nanohole array.

**Figure 4:**
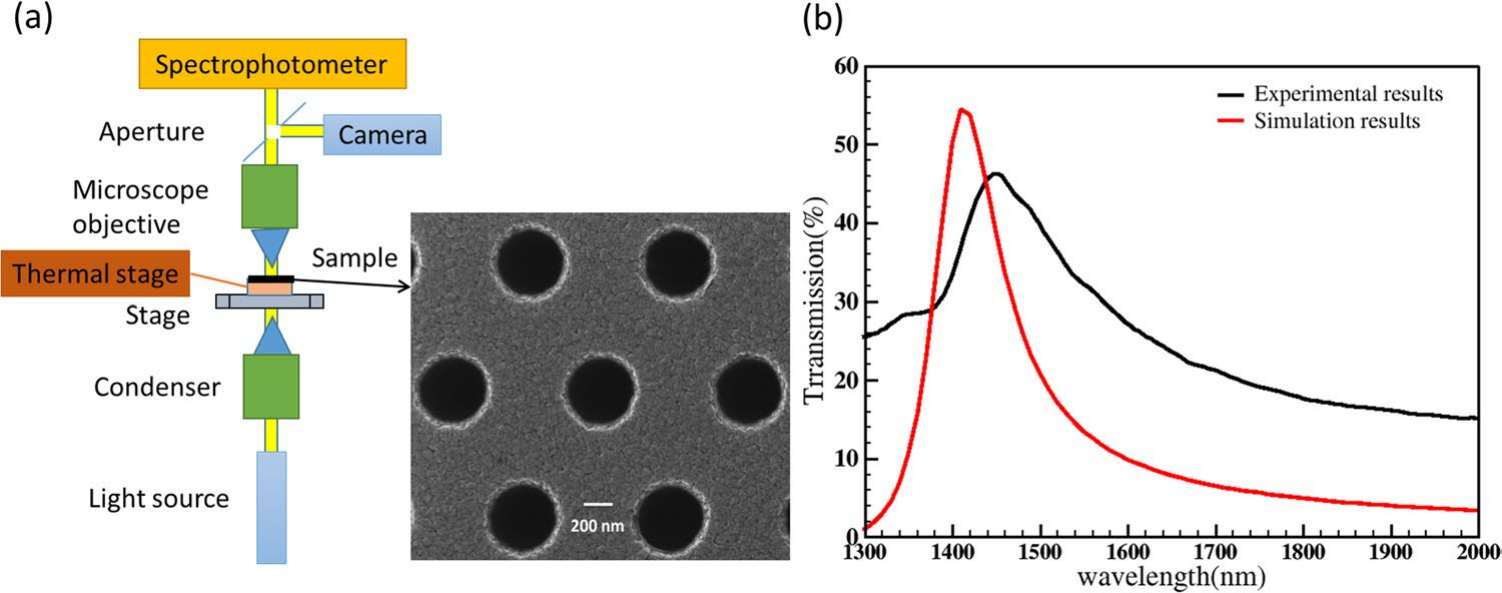
(**a**) Schematic of the experimental setup to measure the optical transmission of the photonic switch with respect to wavelength at different temperatures. The setup consists of CRAIC Technologies 20/30 PVTM micro-spectrophotometer and thermal stage. The light transmitted through the switch is collected by a microscope objective and focused onto the entrance aperture of the spectrophotometer. A beam splitter is used for splitting the light into both camera and the spectrometer. The camera is used for sample alignment. The inset shows the scanning electron microscope image (SEM) of the fabricated aluminium nanohole array with 570 nm diameter and 1010 nm pitch (**b**) Numerically and experimentally obtained transmission spectra from the aluminium nanohole array.

The fabricated photonic switch was characterized using Craic Technologies 20/30 PVTM spectrophotometer and thermal stage as shown in Fig. 4(a). The inset shows SEM image of the hole array in aluminium. As a first step, the nanohole array on aluminium without VO_2_ was characterized to obtain transmission spectrum with respect to wavelength as shown in Fig. 4(b). The experimentally obtained spectrum (black colour) is superimposed with the simulated spectrum (red colour). The experimentally measured peak wavelength is 1447 nm, which is red shifted by 37 nm compared to the peak value of 1410 nm from simulations. The shift is due to fabrication tolerances including slightly larger hole diameter due to undercutting in walls of holes (average hole diameter between top and the bottom is varied between 540 nm to 590 nm due to nanofabrication and measurement tolerances and hence 570 nm is used in simulations). The maximum transmission from the simulation and experiment were 54% and 47% respectively. After this measurement, a VO_2_ layer was deposited on the nanohole array to make the photonic switch. The above transmission measurements were repeated for the hole array with VO_2_ (the photonic switch), and results are discussed in the following section.

**Figure 5:**
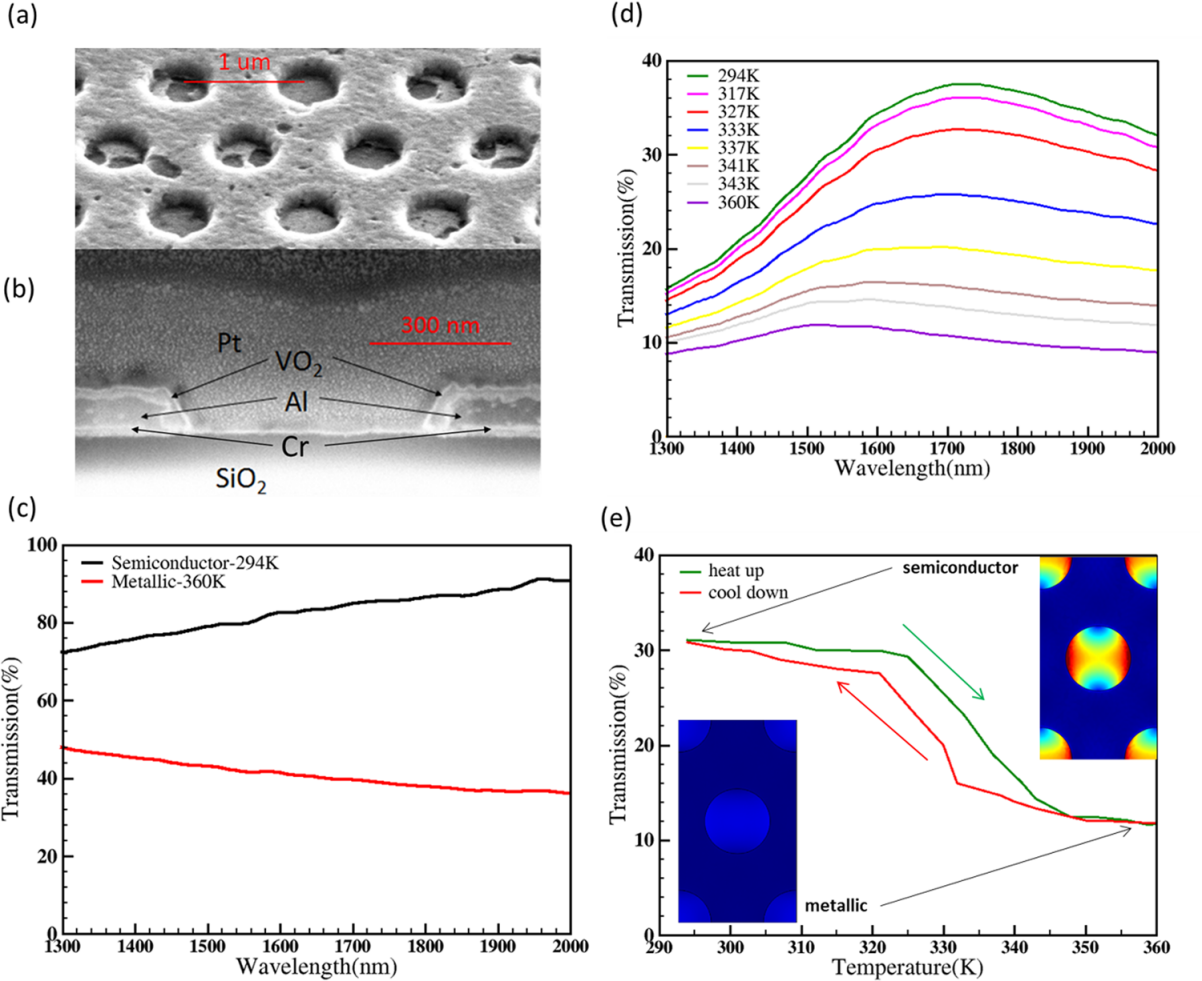
(**a**) SEM image of a tilted top view of the fabricated Al/VO_2_ nanohole array in the photonic switch. (**b**) Cross-section of a single hole obtained using Focused ion beam (FIB) (platinum (Pt) was deposited for contrast). Different materials are marked on the image. From the measurements, Al layer is around 80 nm-thick with 20 nm Cr beneath as adhesion layer, and the VO_2_ layer is 25 nm thick. The diameter of hole is 570 nm based on the average of the top and bottom diameter due to the tilted side wall. (**c**) Experimentally obtained transmission spectrum of the pristine VO_2_ layer with respect to wavelength for both semiconductor (red line) and metallic (black line) phases. (**d**) Experimentally measured transmission of the Al/VO_2_ nanohole array in the photonic switch with respect to temperature (varied from 294 K to 360 K) during which VO_2_ switches from semiconductor phase to metallic phase. (**e**) Experimentally measured optical transmission hysteresis of the photonic switch at 1550 nm during heating and cooling cycle (temperature range: 290 K to 360 K). The two inset images show the simulated E-field intensity distribution in the of VO_2_/Al nanohole array at 1550 nm for metallic and semiconductor phases respectively. The colour legend, the cold tone (blue) to warm tone (red) colour refers to lower intensity to higher intensity (|**E**^2^|).

After the VO_2_ deposition, the cross-section of the photonic switch was taken using FIB to study the distribution of VO_2_ across an area in the sample as shown in Fig. 5(a,b). The SEM images show that VO_2_ covers the sample almost uniformly except creating small patches with no VO_2_ in the holes. These patches slightly reduce the extinction ratio of the device due to light leakage in the metallic phase. This can be avoided with increased VO_2_ thickness at the cost of decreasing transmission percentage. Hence, there exists a trade-off between switching performance and transmission percentage of the device as observed in the simulation results.

In order to find out a suitable temperature range for switching the phase of VO_2_, a 25 nm VO_2_ film on the glass substrate was used. The pristine VO_2_ film was deposited using the same sputtering conditions as the photonic switch. The transmission spectrum of the VO_2_ film was measured. In order to achieve VO_2_ phase transition, the VO_2_ film was heated from room temperature, 294 K (semiconductor phase) to 360 K (metallic phase). Figure 5(c) shows transmission of the VO_2_ film in semiconducting phase (294 K) and metallic phase (360 K). All transmission measured was normalised with respect to the measured area to obtain absolute transmission. In the semiconductor phase, the transmission increases with respect to wavelength, from 76% to 92%, while in metallic phase the transmission drops from 48% to 36%. This result has shown that there is a large difference of 39% in transmission between semiconductor and metallic phase at 1550 nm wavelength and this property can be exploited for making photonic switches and modulators.

Based on the results obtained by testing only VO_2_ sample, the temperature was swept for the photonic switch from room temperature 294 K (semiconductor phase) to 360 K (metallic phase). Figure 5(d) shows transmission spectrum of the photonic switch plotted with respect to different temperatures. As the temperature was increased from 294 K, the maximum transmission has decreased from 37.5% to 11.8% due to the phase change of VO_2_ from semiconductor to a metallic phase. It is also noted that that the peak wavelength of 1725 nm in semiconductor phase (294 K) is slightly blue shifted to 1505 nm when the sample is heated up to metallic phase (360 K). This is due to a low refractive index value of VO_2_ in metallic phase (real part of refractive index reduced from 3.24 to 2.03). This result is also confirmed by simulation of the photonic switch. Transmission of the switch at 1550 nm with respect to temperature was studied by heating the sample from 294 K to 360 K in step of 5 K followed by cooling the switch along the same temperature range as shown in Fig. 5(e). The results show that there is an optical transmission hysteresis curve during heating and cooling cycles which is consistent with the VO_2_ material characters^29^. The fabricated switch has 21% transmission change at C band, 23% transmission change at L band and 26.5% transmission change in U band. The two insets of Fig. 5(e) depicts the simulated electrical field intensity distribution of the photonic switch (VO_2_/Al nanohole array) in semiconductor phase and metallic phase of VO_2_ respectively. The electric field is highly confined in the holes due to the large refractive index contrast between the Al and VO_2_. The maximum transmission peak is 37.5% at 1725 nm (VO_2_ semiconductor phase) and the transmission reduced to 10.5% during the switching of VO_2_ phase to metallic (27% transmission difference).

**Figure 6:**
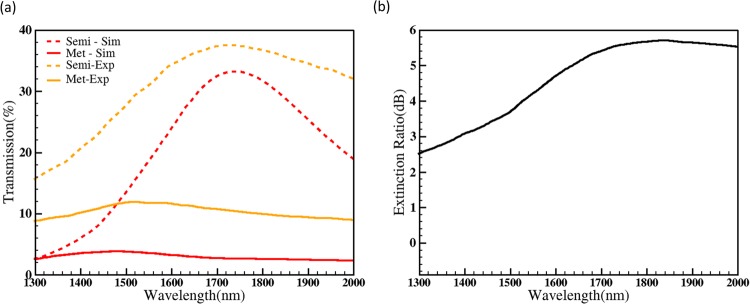
(**a**) Transmission spectra of the photonic switch (570 nm diameter and 1010 nm pitch) for the aluminium thickness of 80 nm, 20 nm Cr and 25 nm VO_2_ for semiconductor phase (dash line) and metallic phases (solid line) from experiments and simulations. (**b**) The extinction ratio of the switch measured from experiments (Extinction ratio: 4.3 dB in C-band, 4.9 dB in L-band and 5.3 in U-band).

From Fig. 4(b), the experimentally obtained peak wavelength of Al nanohole array alone is 47% at 1447 nm. But the peak wavelength of the photonic switch (after depositing VO_2_ in the hole array) is red-shifted to 1725 nm. The red-shift is mainly due to the refractive index of VO_2_ in the nanohole array and fabrication tolerances. Cross-section of a single hole with VO_2_ was taken using Focused Ion Beam after depositing platinum (Pt) in the hole for better contrast as shown in Fig. 5(b). From the cross-section measurements, the thickness of Al, VO_2_ and Cr is measured to be 80 nm, 25 nm and 20 nm respectively, and the hole diameter is taken as 570 nm from the average of the top and bottom diameter due to the undercut. These experimentally obtained values are used in the simulation model of the photonic switch to obtain the transmission spectra of VO_2_ in its semiconductor as well as metallic phase. The simulation results are plotted in Fig. 6(a) and are matching with experimentally measured values. The peak transmission wavelength in semiconductor phase from the simulation is 1735 nm (33.2%) that is close to experimentally obtained the value of 1725 nm (37.5%). From the simulation results, the transmission difference in the photonic switch between the semiconductor and metallic phases of VO_2_ is 16.5% in C band, 24% in L band and 28% in U band which is also close to experimentally obtained values 21% in C band, 23% in L band and 26.5% in U band. Figure 6(b) shows experimentally measured extinction ratio of the photonic switch for different wavelengths of operation. The extinction ratio is 4.3 dB in C band, 4.9 dB in L band and 5.3 dB in U band. The results also show that the switch operates in a 650 nm wavelength range from 1350 nm to 2000 nm with less than 3-dB loss. There is a small light leakage in the photonic switch as shown in Fig. 6(a) due to missing VO_2_ film in some holes (Fig. 5(a)) and also due to 25 nm thickness of VO_2_. The temperature cycling experiment results are provided in supplementary material (S1) with neglected transmission variance. The same heating/cooling process is repeated for each individual measurement and hence the switching effect observed in the device is repeatable.

## Conclusion

We have shown for the first time, a photonic switch using vanadium dioxide as switching material with a hexagonal nanohole array structure, with a maximum 37% transmission at optical communication band. The fabricated switch can achieve 21%, 23% and 26.5% transmission difference in switching with extinction ratio 4.3 dB, 4.9 dB and 5.3 dB in C, L, U-band respectively with a wide operating range over 650 nm. The wide operating range, high transmission and compact device footprint (thickness of 125 nm) give us more flexibility and efficiency in integration and application. In the future, we will further explore the other phase change approaches to increase the response speed of switches, such as using external voltage or laser pumping. The results will have potential applications in developing ultra-compact photonic switches, optical modulators in silicon photonics for optical communications.

### Data availability

All data used in this manuscript are present in the manuscript and its supplementary information.

## Electronic supplementary material

Supporting Information
