# Supplementary material for: A Photonic Switch Based on a Hybrid Combination of Metallic Nanoholes and Phase-change Vanadium Dioxide
Source: Sci Rep. 2018 Jul 23;8:11106. doi: 10.1038/s41598-018-29476-6 (PMC6056514; doi:10.1038/s41598-018-29476-6)
Supplement: Supplementary file 1 — Supporting Information [file 41598_2018_29476_MOESM1_ESM.pdf]

Supporting information for

A Photonic Switch Based on a Hybrid Combination of Metallic Nanoholes and Phase-change Vanadium Dioxide

Miao Sun[1,*], Mohammad Taha[2], Sumeet Walia[2], Madhu Bhaskaran[2], Sharath Sriram[2], William Shieh[1], and Ranjith Rajasekharan Unnithan[1]
[1]Electrical & Electronic Engineering Department, University of Melbourne, Parkville 3010, Australia
[2]Functional Materials and Microsystems Research Group and the Micro Nano Research Facility, RMIT University,
GPO Box 2476, Melbourne, Victoria 3001, Australia
[*]E-mail: miaos1@student.unimelb.edu.au; r.ranjith@unimelb.edu.au

S1. Experimental result for temperature cycling

S1 Shows heating/cooling cycling experimental results of 3 measured transmission spectra of the Al/ VO_2 nanohole array in the photonic switch with respect to Metallic phase and Semiconductor phase (Met/Semi). The first measurement result (showed in Met-1st and Semi-1st) is used in the manuscript as experimental transmission results for the device. The other 2 measurements have undergone the same heating/cooling process (same temperature variation from 294K to 360K). The variance of measured transmission is less than 0.2% and which shows a good repeatability of the switching effect of the device.

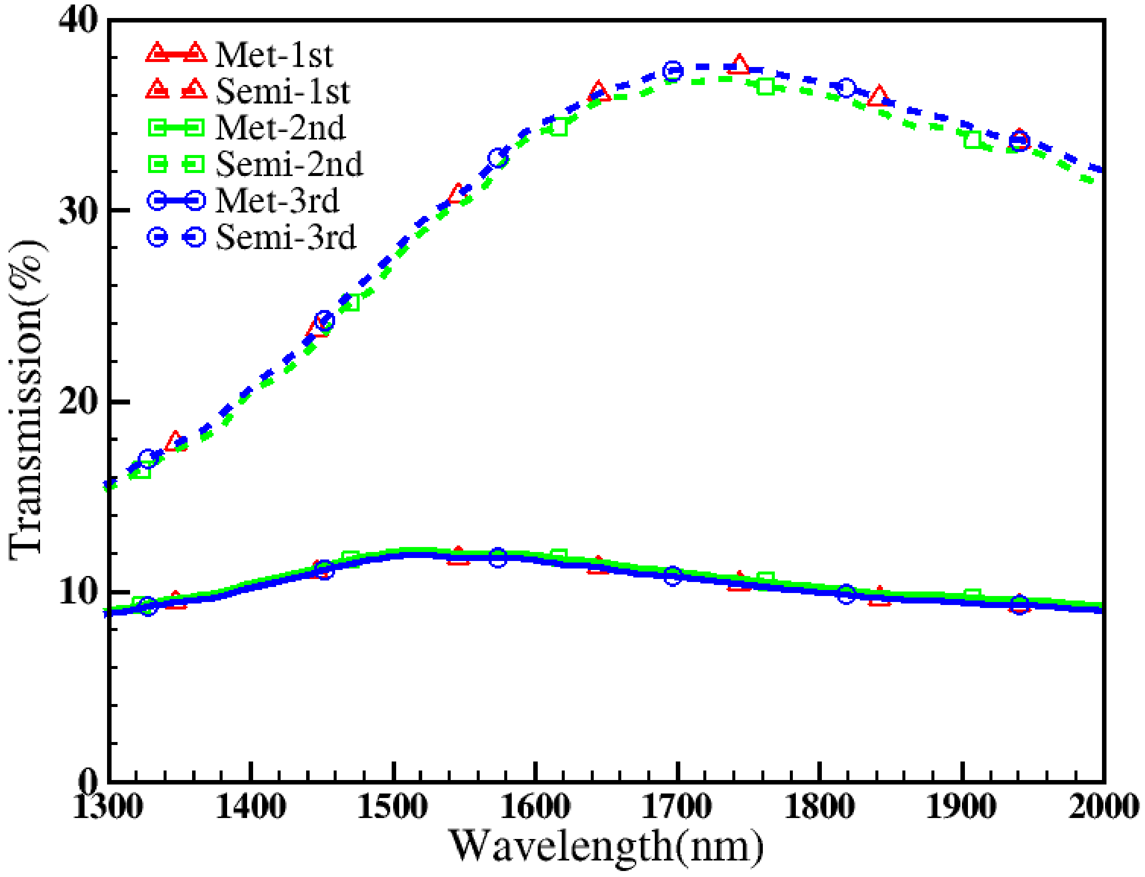

Figure S1 shows heating/cooling cycling experimental results for 3 sets of repeated measurements (represented by the symbols of triangles, squares and circles) of the Al/VO_2 nanohole array in the device. The device has undergone the same heating/cooling process at each individual test, where the transmission spectra of metallic phases (solid line) and semiconductor phases (dash line) are included respectively. The Met-1st and Semi-1st refer to the experimental transmission spectrum of the nanohole array device used in the manuscript.
